# The NeXus data format

**DOI:** 10.1107/S1600576714027575

**Published:** 2015-01-30

**Authors:** Mark Könnecke, Frederick A. Akeroyd, Herbert J. Bernstein, Aaron S. Brewster, Stuart I. Campbell, Björn Clausen, Stephen Cottrell, Jens Uwe Hoffmann, Pete R. Jemian, David Männicke, Raymond Osborn, Peter F. Peterson, Tobias Richter, Jiro Suzuki, Benjamin Watts, Eugen Wintersberger, Joachim Wuttke

**Affiliations:** a Laboratory for Development and Methods, Paul Scherrer Institute, 5232 Villigen-PSI, Switzerland; b ISIS Facility, STFC, Rutherford Appleton Laboratory, Didcot, Oxfordshire OX11 0QX, England; c imgCIF, Dowling College, Shirley, NY 11769, USA; d Lawrence Berkeley National Laboratory, Berkeley, CA 94720, USA; e Spallation Neutron Source, Oak Ridge National Laboratory, Oak Ridge, TN 37831, USA; f Los Alamos National Laboratory, Los Alamos, NM 87545, USA; g Helmholtz-Zentrum Berlin für Materialien und Energie Gmb, 14109 Berlin, Germany; h Advanced Photon Source, Argonne National Laboratory, Argonne, IL 60439, USA; i ANSTO, New South Wales 2234, Australia; j Argonne National Laboratory, Argonne, IL 60439, USA; k Diamond Light Source, Didcot, Oxfordshire OX11 0DE, England; l KEK, Ibaraki 305-0801, Japan; m Swiss Light Source, Paul Scherrer Institute, 5232 Villigen-PSI, Switzerland; n Deutsches Elektronen-Synchrotron DESY, 22607 Hamburg, Germany; o Forschungszentrum Jülich, JCNS at MLZ, 85747 Garching, Germany

**Keywords:** NeXus data format, data exchange, data archiving, platform-independent, HDF5

## Abstract

NeXus is an effort by an international group of scientists to define a common data exchange and archival format for neutron, X-ray and muon experiments. NeXus is built on top of the scientific data format HDF5 and adds domain-specific rules for organizing data within HDF5 files, in addition to a dictionary of well defined domain-specific field names. The NeXus data format has two purposes. First, it defines a format that can serve as a container for all relevant data associated with a beamline. This is a very important use case. Second, it defines standards in the form of application definitions for the exchange of data between applications. NeXus provides structures for raw experimental data as well as for processed data.

## Abstract 2

A description is presented of the NeXus data format for X-ray and neutron scattering and muon spectroscopy.

## 1. Introduction   

Increasingly, major neutron and X-ray facilities have chosen to store data using the NeXus data format. Since the last publication dating from 2006 (Könnecke & NIAC, 2006 ▶), NeXus has undergone substantial refocusing, refinement and enhancement, as described in this paper.

Historically, neutron and X-ray facilities have chosen to store data in a plethora of home-grown data formats. This scheme has a number of drawbacks addressed by NeXus:

(i) It makes the life of travelling scientists unnecessarily difficult as they must deal with multiple files in different formats, file converters and such like in order to extract scientific information from the data.

(ii) An unnecessary burden is imposed on data-analysis software producers to accommodate many different formats.

(iii) The whole idea of open access to data is sabotaged if the data are in a format which cannot be easily understood.

(iv) Scientific integrity is jeopardized if the data cannot be understood or important elements are missing.

(v) Modern high-speed detectors produce data at such a high rate that many older single-image storage schemes have become impractical and an efficient container format is mandatory.

The primary necessity for a data format is to define a physical file format: how are the data written to disk? Rather than invent yet another format, NeXus chose HDF5 (HDF Group, 2014 ▶ *a*) as the physical file format. HDF5 is a binary file format in the public domain and is well supported by both commercial and free software tools. It is efficient, self-describing and platform-independent.

NeXus data files are built using basic HDF5 storage elements: data groups (like file-system folders), data fields (such as strings, floats, integers and arrays), attributes (additional descriptors of groups and fields) and links (like file-system links). NeXus is implemented in terms of these basic storage elements.

NeXus adds to HDF5:

(i) Rules for organizing domain-specific data within an HDF5 file, most notably a data hierarchy for arranging data in the file.

(ii) A link structure to enable quick default visualization

(iii) A dictionary of documented domain-specific fields expressed in the form of base classes.

(iv) Definitions of standards that can be validated in the form of application definitions.

The development of NeXus is overseen by a committee, the NeXus International Advisory Committee (NIAC) (NIAC, 2014*b* ▶).

## 2. General principles   

The authors of data-acquisition and instrument-control software are encouraged to generate exactly one NeXus container file per measurement (a measurement is either a data accumulation under fixed conditions or a scan). This file includes not only the detector and monitor data, but also metadata, information on the state of the beamline, parameter logs and more. Authors of data-reduction and data-analysis software can use NeXus to store processed data along with metadata and a processing log.

NeXus can be used for many different experimental techniques and at different levels of data processing. For each of these different applications, a specific subset of the standardized NeXus entities (data groups and fields) is needed. The minimum set of fields for a particular use case is standardized in the NeXus application definitions (§6). The minimum set can always be enhanced by adding additional fields from the NeXus base classes (§5).

As a container format, NeXus allows files to be extended at any moment by additional content, including NeXus base classes, HDF5 groups and HDF5 data sets. Because HDF5 provides full read–write access to the file, such changes can be made in an existing NeXus file without the need to write a complete new file.

The combination of a well defined hierarchy of groups with a comprehensive and well documented dictionary of data and metadata names ensures that NeXus files are self-describing. It should be possible for another scientist to understand the contents of a NeXus file without consulting documentation specific to any one facility or beamline. By enabling the storage of comprehensive metadata, the NeXus format facilitates the sharing of data between collaborators and long-term data curation.

## 3. Data file hierarchies   

NeXus uses a group hierarchy for arranging data in an HDF5 file. Data files may contain raw data or processed data or both, each of which has a hierarchy.

### 3.1. Raw data file hierarchy   

**Figure 1:**
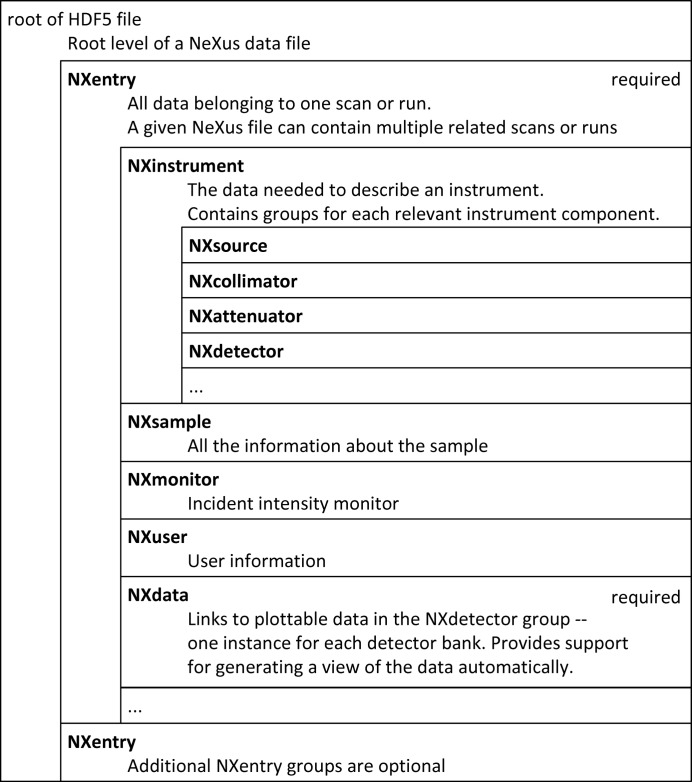
An overview of the structure of a NeXus raw data file. Note that only a small part of this structure (the first NXentry group and the first NXdata group) is actually required. The other content is optional.

The major focus of NeXus has been the recording of raw experimental data, *i.e.* information taken directly from the experimental equipment or processed only as required to provide physically meaningful values. The NeXus raw data file hierarchy is the consequence of some practical considerations. An overview of the NeXus data file structure for raw experimental data is shown in Fig. 1 ▶.

When looking at a beamline it is easy to discern different components: beam optic components, sample position, detectors and such. It is quite natural to replicate this physical separation with a logical arrangement of storing the data from each component in a separate group. This approach explains the list of beamline components in the NXinstrument group presented in Fig. 1 ▶. As there can be multiple instances of the same kind of equipment, such as slits or detectors, in a given beamline, it becomes necessary to add type information to the group name. This type information, the NeXus class name, is provided by an HDF5 attribute. By convention, NeXus class names start with the prefix NX. Each NeXus group describing a beamline component contains further groups and fields describing the component. A field can contain a single number, a text string or an array, as appropriate for the data to be described.

The need to be able to store multiple related scans or runs in the same file, or to capture a complete workflow in a file, causes the beamline component hierarchy to be pushed one level deeper into an NXentry group in the hierarchy. The NXentry group thus represents one scan or run (or a processed data entry, as will be discussed later). The NXentry group also holds the experiment metadata, such as the date and time at which it was performed.

In the course of the evolution of NeXus, the decision was taken to move NXmonitor out of NXinstrument to the higher hierarchy level of NXentry, in order to facilitate quick inspection by humans.

To enable a simple default visualization, an NXdata group must be provided at the NXentry level. It contains information about plot axes and links to the data (which typically reside in the NXdetector group). Links are supported by HDF5 and work like hard links in the Unix file system.

A special base class, NXcollection, exempts its contents from validation and thereby allows inclusion of any data in arbitrary non-NeXus formats.

#### 3.1.1. Multiple-method instruments   

**Figure 2:**
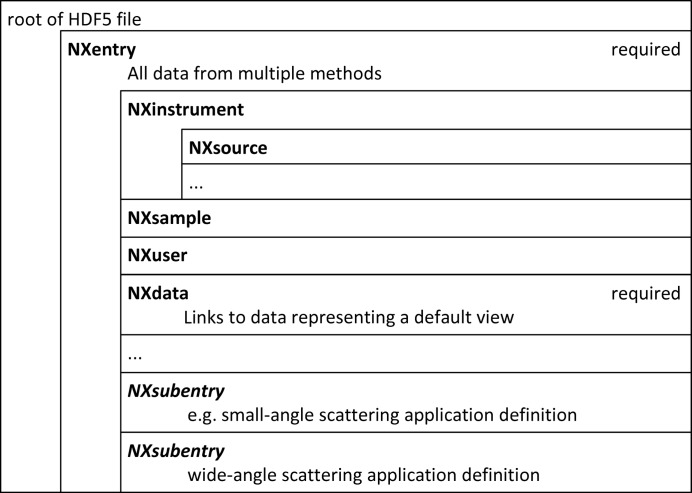
An overview of the structure of a NeXus raw data file for an instrument with multiple methods.

Particularly at X-ray sources, some instruments offer multiple techniques that can be used in parallel. For example, small-angle scattering and powder diffraction can be measured simultaneously at a SAXS/WAXS beamline. We recommend storing the data from all methods in one file in a single NXentry hierarchy (Fig. 2 ▶). All information from all detectors, logs and such like are collected in this one NXentry group to keep the data together. Information that is particular to one experimental technique is linked to an NXsubentry. The NXsubentry follows the hierarchy of NXentry, but it will typically only link to the data required by the application definition for the specific experimental technique. The point of this scheme is that both human and computerized users can easily locate method-specific data while maintaining the full view of the experiment.

#### 3.1.2. Scans   

Scans come in all shapes and sizes. Almost anything can be scanned against anything. An additional consideration is that, in practice, the final number of scan points in the scan cannot be known in advance, since it is possible that a scan may be interrupted or terminated before its planned number of observations. Thus, it is a challenge to standardize a scan. NeXus addresses this challenge through the use of the HDF5 ‘unlimited dimensions’ feature and additional conventions as described below. With the HDF5 unlimited dimensions feature, one axis of the data is allowed to expand without limit. Thus, the size of a data array does not need to be declared in advance. Data can be appended to an array along the unlimited dimension as required.

Scans are stored in NeXus following these two conventions:

(i) Each variable varied or collected in the scan is stored at its appropriate place in the NeXus beamline hierarchy as an array. The first dimension of the array is the scan axis. This is the unlimited dimension in the implementation, and data at each scan point are appended to the array.

(ii) The NXdata group holds links to all the variables varied or collected during the scan. This creates something equivalent to, or better than, the tabular representation people are accustomed to for scans. The detector data can be plotted against any scanned parameter, as well as against everything that was deemed worth recording in addition to that. The necessary data are all gathered together in the NXdata group, either directly or *via* links, so that other groups do not normally have to be searched to do this plotting.

NeXus also allows multi-dimensional scans. This makes it very simple to produce meaningful slices through data volumes, even with NeXus-agnostic software (*e.g.* *HDFView*; HDF Group, 2014 ▶ *b*).

### 3.2. Processed data file hierarchy   

**Figure 3:**
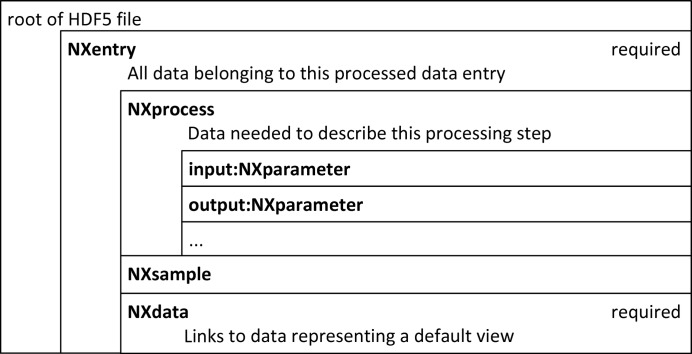
An overview of the structure of a NeXus processed data file.

At the request of the user community, NeXus has created a simplified structure for storing the results of data processing, be it reduction or analysis. An overview of the NeXus structure for processed data is given in Fig. 3 ▶.

The hierarchy is much reduced, as it is not important to carry all experimental information into the data reduction. In contrast with the raw data file structure, NXdata in the processed file structure is the place to store the results of the processing, together with its associated axes if the result is a multi-dimensional array.

In addition to the NXdata and NXsample groups, the NXprocess group provides a structure to store details about the processing, such as the program (or programs) used, its version, the date of processing and other metadata. The NXprocess group can hold additional NXparameter groups, which are containers for storing the input and output parameters of the program used to perform the processing.

## 4. Coordinate systems, positioning of components and further rules   

For data reduction, it is often necessary to know the exact position and orientation of beamline components. The first thing needed is a reference coordinate system. NeXus has chosen to use the same coordinate system as the neutron beamline simulation software *McStas* (Willendrup *et al.*, 2004 ▶).

For describing the placement and orientation of components, NeXus stores the same information as is used for the same purpose in the crystallographic information file (CIF; Hall & McMahon, 2005 ▶). CIF (and NeXus) stores the details of the translations and rotations necessary to move a given component from the zero point of the coordinate system to its actual position. As coordinate transformations are not commutative, the order of transformations must also be stored.

The reader is directed to the NeXus manual for further rules on the handling of axes, units and special cases of data storage.

## 5. Base classes   

As can be seen from the discussion of the NeXus file hierarchy, NeXus arranges data in groups, which have a type descriptor and a NeXus base class name associated with them. Technically, the class name is the value of the HDF5 attribute NX_class. The term ‘base class’ is not used in the same sense as in object-oriented programming languages; in particular, there is no inheritance. The NeXus base classes provide a comprehensive dictionary of terms that can be used for each class. The terms in the dictionary comprise concepts and names common to the topic of the base class. The expected spelling and definition of each term are specified in the base classes. It is neither expected nor required to provide all the terms specified in a base class. These terms designate data fields that can be stored within a group. A data field can have a simple type (like integer, float, date/time, binary), or it can be a NeXus subgroup. The base class definition also contains informal annotations about the semantics of each field. Terms with other names are permitted but might not be recognized by standard software.

At base class level, NeXus has no mechanism to mark some fields as obligatory. All allowed fields are optional. Which of them are written into data files must be decided according to application needs. These decisions can be standardized in the form of application definitions (see below, §6).

The NeXus base classes are encoded in the NeXus Description Language (NXDL) (NIAC, 2014 ▶ *c*). NXDL is just another form of an XML file that specifies the content of a NeXus base class. NXDL files may be parsed either by humans or by software and may be validated for syntax and content. The NXDL files are used to validate the structure of NeXus data files. Java source code for a GUI (graphical user interface) tool has been prepared (NIAC, 2014 ▶ *d*) to perform such validation.

## 6. Application definitions   

An application definition, expressed in NXDL, specifies a data structure for a given application domain, such as a scientific technique or a specific type of instrument. The data structure consists of a hierarchy of NeXus groups, for each of which a minimum content is specified. Application definitions are therefore different from base class definitions, which specify a comprehensive dictionary of terms that can be used.

Historically, an application definition addressed just one type of instrument, like an X-ray reflectometer or a direct-geometry neutron time-of-flight spectrometer. Thus, application definitions were originally named ‘instrument definitions’. However, as NeXus can also be used for processed data, such as a tomographic reconstruction or a dynamic scattering law *S*(*Q*, ω), the more generic term ‘application definition’ has been adopted.

## 7. Contributed definitions   

The process of drafting and ratifying application definitions is ongoing (see also below, §8). Currently, scientists representing both the NIAC and the IUCr Committee on the Maintenance of the CIF Standard have nearly finished with a NeXus application definition for macromolecular crystallography (MX). CBFlib (Bernstein & Ellis, 2005 ▶; http://sourceforge.net/projects/cbflib and http://www.bernstein-plus-sons.com/software/CBF/) is being extended to work with the NeXus MX format, and this work will be published in another paper. Work on another NeXus application definition for reduced small-angle scattering data is also in progress (canSAS, 2014 ▶) by scientists representing canSAS, NeXus and the IUCr Commission on Small-Angle Scattering.

All such proposals from the scientific community to extend NeXus with new application definitions and base classes are added to NeXus, initially, as contributed definitions, either in incubation or as a special case not for general use. The NIAC (see *Governance*, below) is charged to review any new contributed definitions and to provide feedback to the authors before ratification and acceptance.

## 8. Governance   

The development of NeXus is overseen by the NIAC (NIAC, 2014 ▶ *b*). The NIAC seeks a balanced representation of the international community. Most major neutron, X-ray and muon facilities have appointed delegates. Other facilities are invited to join.

The NIAC reviews any proposed amendments to the NeXus base classes and application definitions, and holds online votes to ratify changes. A great number of candidate NeXus application definitions exist which were derived from our understanding of the technique described. For each of these, the NeXus team seeks community approval.

## 9. Uptake of NeXus   

NeXus is already in use as the main data format at many facilities, including SOLEIL (France), Diamond (UK), SINQ (Switzerland), SNS (USA), Lujan/LANL (USA) and KEK (Japan). Other facilities including ISIS (UK), DESY (Germany) and the μSR (muon spin rotation/relaxation/resonance) community are in the process of moving towards NeXus as their data format. At LBNL (USA), NeXus is currently being adapted for X-ray free-electron laser (XFEL) serial crystallographic data. APS (USA) is storing some of its data collection using NeXus. The *EPICS* (Rivers, 2014 ▶) area-detector software has a plug-in to write acquired images to NeXus data files. Also, some commercial manufacturers of area detectors now write acquired images to NeXus data files.

The adoption of NeXus has taken some time. The reason is that NeXus is often chosen whenever a facility starts operation or undergoes major refurbishment. For those facilities where there is an existing and working pipeline from data acquisition to data analysis, the resources are usually lacking to move towards NeXus as the only data file format.

This is reflected in the experience of the muon community. For the ISIS source, the move to a Windows PC-based data-acquisition system in 2002 required a new data format, providing an ideal opportunity to exploit the emerging NeXus standard (Flannery, 2003 ▶). In contrast, sources at PSI (Switzerland), TRIUMF (Canada) and KEK continue to make good use of existing formats and software. More recently, funding from the EU has enabled the community to develop the application definition as a common exchange format for muon data (Cottrell *et al.*, 2012 ▶).

Whether used as the main or an intermediate format, users are able to produce compatible NeXus files from data written at all these facilities, enhancing the uptake of NeXus within the community.

## 10. Backwards compatibility   

Historically, NeXus supported reading and writing data files in HDF4, HDF5 and XML formats by use of the NeXus Application Programming Interface (NeXus API or NAPI). The NAPI is still available, but is frozen except for bug fixes. After consultation with the community, the currently recommended use of NeXus is solely in terms of the HDF5 file format, using standard HDF5 tools. This is expected to remain the basis for NeXus software development and file creation in the future.

## 11. Summary   

NeXus has matured considerably over the past ten years and is now in use in many facilities. NeXus is flexible enough to accommodate a wide variety of instruments and scientific applications, yet efficient enough to handle the data coming from modern high-speed detectors. More information, including a full manual in PDF format, can be found on the project web site (NIAC, 2014 ▶ *a*). Members of the NIAC (NIAC, 2014 ▶ *b*) always welcome correspondence concerning the development of the NeXus data format.
